# Subthreshold Micropulse Laser for Long-Lasting Submacular Fluid after Rhegmatogeous Retinal Detachment Surgery

**DOI:** 10.18502/jovr.v17i3.11577

**Published:** 2022-08-15

**Authors:** Giulia Esposti, Pier Luigi Esposti, Francesco Costantino, Dario Zappalà, Antonio Pinna, Mario Fruschelli

**Affiliations:** ^1^Studio Oculistico Esposti, Siena, Italy; ^2^Graduate School of Ophthalmology, University of Siena, Siena, Italy; ^3^Department of Medical, Surgical, and Experimental Sciences, Ophthalmology Unit, University of Sassari, Sassari, Italy; ^4^Dipartimento di Scienze Mediche Chirurgiche e Neuroscienze, University of Siena, Siena, Italy

**Keywords:** Optical Coherence Tomography, Retinal Pigment Epithelium, Rhegmatogenous Retinal Detachment, Subretinal Fluid, Subthreshold Micropulse Laser

## Abstract

**Purpose:**

To assess the safety and efficacy of subthreshold micropulse laser (SML) photo-stimulation in the management of persistent subfoveal fluid (PSF) after surgery for rhegmatogenous retinal detachment (RRD).

**Methods:**

In this pilot study, 11 eyes of 11 patients (8 men, 3 women) with long-lasting (12–18 months) PSF after surgery for RRD were evaluated before and after photostimulation with subthreshold micropulse yellow laser. Ophthalmic examination included best-corrected visual acuity (BCVA), Amsler grid test, ophthalmoscopy, autofluorescence (AF), and optical coherence tomography (OCT) with measurement of central point foveal thickness (CPFT). Primary outcome was subfoveal fluid resolution and secondary outcome was BCVA improvement.

**Results:**

The mean CPFT and BCVA were, respectively, 436.8 ± 28.8 μm and 0.25 ± 0.1 µm decimal equivalent (DE) before photostimulation and 278 ± 54.4 μm and 0.57 ± 0.2 µm DE after photostimulation, a statistically significant difference (*P* < 0.001). Nine (81.8%) eyes showed improved BCVA, disappearance of macular detachment on ophthalmoscopy, reduced retinal pigment epithelium distress on AF, and restored macular profile with no neuroretinal alterations on OCT scans.

**Conclusion:**

Although PSF after RRD surgery is often a self-limiting disease, our results suggest that SML photostimulation may be effective and safe in patients with clinically significant long-lasting PSF. Larger case–control studies are necessary to confirm these results.

##  INTRODUCTION

Retinal detachment occurs when the neuroepithelium separates from the underlying retinal pigment epithelium (RPE) and fluid accumulates in the subretinal space.^[1]^ Non-traumatic rhegmatogenous retinal detachment (RRD) in phakic eyes has an incidence of about 1:10.000/year. Surgery is the gold standard treatment for RRD. There are three main types of RRD surgery: episcleral, intrascleral, and vitreoretinal. Regardless of the surgical technique chosen, the surgical goals are to identify and close all the breaks and reduce vitreoretinal traction. Closure of the breaks occurs when the edges of the retinal break are brought into contact with the underlying RPE. Persistence of subretinal fluid (SRF) after closure of the retinal breaks is much higher after scleral buckle surgery than after vitrectomy (55% vs 15%).^[2,3]^ Persistence of SRF may impair visual recovery and be responsible for poor central vision, metamorphopsia, and loss of depth perception. SRF is usually self-limiting,^[4]^ but may occasionally become chronic and damage photoreceptors with permanent vision loss.^[5]^ Several authors have suggested possible explanations for SRF formation after RRD surgery, but without conclusive evidence.^[6]^

RPE plays a crucial role in subretinal fluid resorption.^[1,7]^ However, if RPE function is insufficient to restore the normal retinal anatomy and the fluid accumulates in the macular area, persistent subfoveal fluid (PSF) with metamorphopsia and visual loss may occur.^[4,5]^

The presence of PSF after episcleral or vitreoretinal surgery for RRD is a relatively uncommon, self-limiting complication.^[2,3]^ The management of PSF following repair of RRD has typically been observation, because this condition often resolves spontaneously. In refractory cases, vitrectomy with gas tamponade may be considered.^[5]^ Recently, Koinzer et al have suggested a noninvasive approach by selective retina therapy, a new laser technology using a train of µs-laser pulses to selectively damage RPE cells.^[8]^

The aim of this pilot study was to investigate the efficacy and safety of subthreshold micropulse laser (SML) photostimulation^[9]^ in 11 eyes of 11 patients with symptomatic, long-lasting PSF after successful RRD surgery.

##  METHODS

This study was conducted in compliance with the tenets of the Declaration of Helsinki for research involving human subjects. Institutional ethics review board approval was obtained. Each participant received detailed information and provided written consent.

A total of 11 patients (8 men, 3 women; mean age 54.3 ± 5.5 years), who had undergone RRD surgery at our ophthalmology unit between September 2012 and October 2016, were recruited in this pilot study. Eight patients had episcleral surgery only, whereas three had episcleral + vitreoretinal surgery. All patients complained of long-lasting (range: 12–18 months, mean: 14.72 months) distorted vision and difficulty in reading after surgery. Common diagnosis was macular detachment due to PSF after RRD surgery. All subjects underwent a full ophthalmic examination, including measurement of best-corrected visual acuity (BCVA), funduscopic evaluation under pharmacological mydriasis (Volk 90D no contact slit-lamp lens, Volk Opticals, Mentor on the Lake, OH, USA), Amsler grid test, central point foveal thickness (CPFT) determined by structural optical coherence tomography (OCT) (RTVue-100Ⓡ, Optovue Inc. Fremont, CA, USA), and autofluorescence (AF) with ultra-widefield imaging (Daytona ${}^{TM}$ , Optos, Marlboruogh MA, USA).

A single session of SML photostimulation with yellow diode laser (IQ 577 ${}^{TM}$ , Iridex Corporation, Mountain View, CA, USA) was performed. The following protocol was used: mydriasis with topical 1% tropicamide, anesthesia with 4% benoxinate eye-drops, application of an Area CentralisⓇ contact lens (field of view 70/84º, image magnification 1.06x, laser spot 0.94%, Volk Opticals, Mentor on the Lake, OH, USA), and SML photostimulation. Laser parameters were set as follows: confluent spots at 70% of the minimum power necessary to obtain retinal whitening in micropulse mode, 100 µm diameter (100 µm x 0.94 laser factor = 94 µm), 200 ms duration, 5% operating duty cycle. The power used was 440 to 530 mW (mean: 466.3 mW). Laser spots were performed on the entire edematous area; then, tobramycin-dexamethasone eye-drops were administered twice a day for 15 days. Patients were re-examined after one, two, and three months and then every three months for 12–36 months. Each follow-up evaluation included BCVA, ophthalmoscopy, Amsler grid test, AF and OCT. The raw data were reported in an Excel sheet and then transferred to the Statistical Package for the Social Sciences, version 21 (IBM Corporation, Armonk, NY, USA) for analysis. Pre- and posttreatment BCVA and CFT were analyzed using paired student's *t*-test.

**Figure 1:**
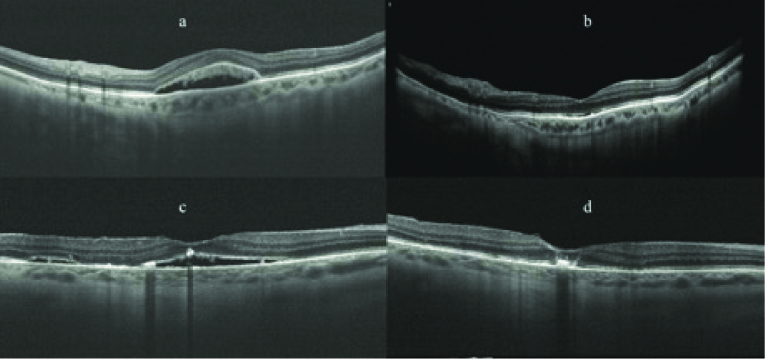
Cases 1 and 2: OCT before SML treatment showed an optically empty macular space between RPE and neuroepithelium with granular deposits adhering to the outer photoreceptor segments (a & c). After photostimulation, PSF was undetectable by OCT and granular deposits were unchanged (b & d).

**Figure 2:**
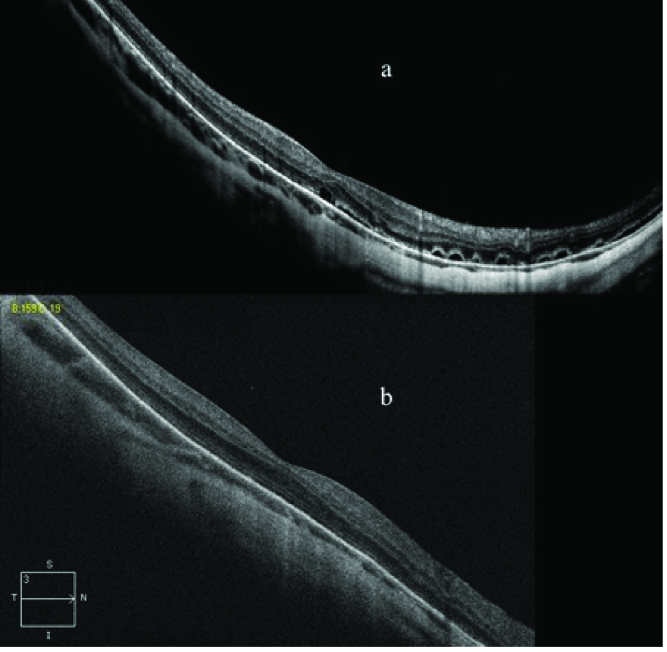
Case 5. OCT in a myopic patient with evidence of multiple areas of serous detachment of the neuroepithelium (a). Six months after SML photostimulation there are no detectable areas of detachment (b).

**Table 1:** Details of patients who underwent subthreshold micropulse laser (SML) photostimulation for persistent subfoveal fluid (PSF) after rhegmatogenous retinal detachment surgery.


	**Sex/Age**	**Episcleral surgery (E) or vitrectomy (V)**	**Time between surgery and photostimulation (months)**	**Central point foveal thickness pre photostimulation**	**Visual acuity pre photostimulation**	**Power, mW**	**Number of spots**	**Central point foveal thickness post photostimulation**	**Visual acuity post photostimulation**	**Follow-up (months)**	**Time between photostimulation and resolution of PSF (months)**
			**(decimal equivalent)**		**(decimal equivalent)**	
	m/50	E	18	503	0.025	500	400	219	0.3	24	3
	m/55	E	18	402	0.3	480	400	245	1	21	3
	f/45	E	16	443	0.2	530	766	246	0.7	33	2
	m/60	E+V	12	423	0.2	440	680	271	0.7	12	6
	m/52	E	16	438	0.1	490	450	270	0.6	36	6
	m/63	E	14	440	0.2	420	450	265	0.4	12	2
	f/55	E+V	12	420	0.3	490	430	365	0.3	15	NR*
	f/57	E	12	466	0.3	450	520	270	0.6	15	1
	m/51	E	14	421	0.4	440	460	256	0.7	18	6
	m/49	E	12	444	0.3	450	530	400	0.4	21	NR*
	m/60	E+V	18	405	0.4	440	490	251	0.6	15	1
Mean ± SD	54.3 ± 5.5 yrs	14.73 ± 2.57	436.82 ± 28.77	0.25 ± 0.11	466 ± 34	506 ± 116	278 ± 54.4	0.57 ± 0.21	20 ± 8	3.3 ± 2.12
	
	
*No resolution of PSF											

##  RESULTS

The mean CPFT before and after photostimulation were 436.8 ± 28.8 and 278 ± 54.4 μ m, respectively, a statistically significant difference (*P* < 0.00001). The mean BCVA before and after photostimulation were 0.25 ± 0.1 and 0.57 ± 0.2 decimal equivalent (DE), respectively, again a statistically significant difference (*P* < 0.0001). There was BCVA improvement in 10 (90.9%) eyes, whereas vision remained unchanged in patient 7, who had undergone episcleral + vitreoretinal surgery. There was a mean decrease in CPFT of 149.8 μm (34.2%). In all cases, subfoveal fluid was ophthalmoscopically undetectable, Amsler grid test was negative, and OCT scans showed a marked reduction of the optically empty space between RPE and neuroepithelium. Time between photostimulation and PSF resolution was one to six months (mean: 3.3 ± 2.1 months), but in two cases (case 7, who had undergone episcleral + vitreoretinal surgery, and case 10, who had undergone episcleral surgery) the treatment was ineffective [Table 1]. No RPE changes were detected on OCT and AF during the follow-up period. All patients underwent only one session of SML photostimulation. In cases 1 and 2, granular deposits adherent to the outer photoreceptor segments remained unchanged after photostimulation [Figure 1]. Case 5 had diffuse myopic degeneration, positive Amsler grid test, AF evidence of apparently normal posterior pole, but OCT evidence of multiple areas of serous detachment of the neuroepithelium. Six months after photostimulation, Amsler grid test was negative and OCT confirmed disappearance of the neuroepithelial detachment, whereas ophthalmoscopy and AF pictures remained unchanged [Figure 2]. In the remaining cases, no remarkable photostimulation-induced alterations were found**. **No adverse effects related to SML photo-stimulation were recorded.

##  DISCUSSION

In this pilot study investigating the efficacy and safety of SML photostimulation in eyes with symptomatic, long-lasting PSF after successful RRD surgery, we found that the mean CPFT and BCVA improved significantly after SML treatment. Overall, 9 (81,8%) eyes out of 11 showed improved BCVA, disappearance of macular detachment on ophthalmoscopy, reduced retinal pigment epithelium distress on AF, and restored macular profile with no neuroretinal alterations on OCT scans.

To maintain adhesion to RPE, the neuroepithelium relies on a variety of active and passive mechanisms (hydrostatic and choroid oncotic pressure).^[1]^ RPE, an active pump, is responsible for the main mechanism, draining fluid toward the choroid at a rate of 0.1–0.3 ml/h/mm ${}^{2}$ and accounting for 70% of transepithelial fluid movement. This polarized monocellular layer maintains ionic gradients by means of the Na ${}^{+}$ /K ${}^{+}$ pump and aquaporin-1 channels at the apex of the plasma membrane.^[10]^ Other adhesion factors between the retina and choroid include apical interdigitations of the RPE and a mucopolysaccharide matrix, known as interphotoreceptor matrix, in the subretinal space.^[11,12]^ RPE activity is therefore essential for adhesion of the neuroepithelium and resorption of residual subretinal fluid after RRD surgery.

We used SML photostimulation with the aim to reactivate RPE and favor resorption of residual subfoveal fluid. Micropulse diode laser has the appropriate characteristics for this purpose.^[13,14]^ A continuous laser emits a constant flow of energy, albeit with very short exposure times, whereas in micropulse mode the emission is fractionated into a train of brief impulses, the duration (on) and interval (off) of which can be varied by the operator. Brief duration limits the time during which laser-induced heat diffuses into adjacent tissues (neuroepithelium); a longer interval allows more time for tissue cooling.^[15]^ This type of laser emission is therefore devoid of endpoint retinal “whitening”.^[7,9]^ No laser trace is left on the retina, undetectable by ophthalmoscopy during and after treatment, or by AF or OCT scans after treatment.^[16,17]^ The choice of wavelength 577 nm is justified by the interaction of the laser with the tissue and conversion of laser energy into heat. The three main chromophores that absorb light are melanin (the most efficient, located in the RPE and choroid, where energy is converted into heat), hemoglobin (the highest absorption coefficient of 577-nm radiation is found in oxyhemoglobin of the choriocapillaris), and xanthophyll (found in the inner and outer plexiform layers of the macula, where light of this wavelength is only minimally absorbed). All this favors laser selectivity for RPE, sparing the internal layers of the neuroretina. Certain wavelengths can penetrate deeper layers, regardless of the uneven distribution of melanin. The different distribution of the three chromophores ensures a uniform effect of the laser in eyes with little or irregular fundus pigmentation.^[18]^ Subthreshold micropulsed laser has beneficial intracellular biological effects without any visible damage during or after treatment. Photostimulation reactivates RPE cellular activity,^[19,20]^ with up- and downregulation of various RPE factors stimulating fluid resorption,^[21,22]^ such as overexpression of stromal cell-derived factor 1 (SDF1), which attracts stem cells and restores the normal function of RPE by renewing dead epithelial and endothelial cells.^[23,24]^ Photothermal stimulation induces the expression of heat shock protein (HSP), which normalizes the levels of cytokines and reduces chronic inflammation.^[19]^

PSF after retinal surgery for RRD is a relatively uncommon and self-limiting condition, which can occasionally cause major visual loss. The management of PSF following repair of RRD has typically been observation. In refractory cases, vitrectomy with gas tamponade may be considered. In our small case series, SML photostimulation with yellow diode laser yielded a statistically significant improvement in CPFT (*P* < 0.00001) and BCVA (*P* < 0.0001). Our results are consistent with the observation reported by Landa.^[25]^ Overall, these findings suggest that SML photostimulation may be an effective, noninvasive, and safe approach for PSF management.

As this was a pilot study, the number of patients was small and sample size planning to ensure an adequate power was unnecessary. The main limitation of our investigation is that it was not a randomized prospective case–control study.

As there is no definitive proof of efficacy of steroids or oral diuretics in this condition,^[26]^ multicentric randomized studies comparing SML photostimulation with simple observation are warranted to establish whether, or not, SML can be considered as a potential, noninvasive alternative for the treatment of PSF after RRD surgery.

##  Ethical Approval

This article does not contain any study with animals performed by any of the authors. All procedures performed in this study involving human participants were done in accordance with the ethical standards of the institutional and/or national research committee and with the 1964 Helsinki Declaration and its later amendments or comparable ethical standards. Informed consent was obtained from all individual participants included in the study.

##  Financial Support and Sponsorship 

This research did not receive any specific grant from funding agencies in the public, commercial, or not-for-profit sectors.

##  Conflicts of Interest

The authors report no conflicts of interest. The authors alone are responsible for the content and writing of the paper.
